# Impact of International Travel on Clinical Outcomes of Mpox Cases in Jeddah, Saudi Arabia: A Retrospective Cohort Study

**DOI:** 10.7759/cureus.113437

**Published:** 2026-07-27

**Authors:** Faisal A Alzahrani, Fatemah Alzaghabi, Abdullah Al-Garni, Ibrahim Asiri, Abdulelah A Alzahrani

**Affiliations:** 1 Public Health, King Fahad Hospital, Al Baha, SAU; 2 Western Region Branch, Public Health Authority, Jeddah, SAU; 3 Faculty of Medicine, Al Baha University, Al Baha, SAU

**Keywords:** clinical outcomes, hospital length of stay, international travel, jeddah, locally acquired infection, monkeypox, mpox, retrospective cohort study, saudi arabia

## Abstract

Background: Saudi Arabia is a global hub for air travel and mass gatherings; the first case of mpox in the Kingdom was linked to international travel. It is not clear whether travel-related mpox cases differ from locally acquired cases in how they present or in their outcomes in Jeddah, a major gateway city. We compared the clinical characteristics and outcomes of travel-related and locally acquired mpox cases.

Methods: This was a retrospective cohort study including 232 lab-confirmed cases in Jeddah. Cases were taken from the Health Electronic Surveillance Network (HESN) and hospital records of the first and second health clusters in Jeddah. They were grouped as travel-associated if international travel occurred within 21 days before symptom onset or the first positive test result, or as locally acquired. Then, we compared them by demographics, clinical features, disease severity, and outcomes, including hospitalization, ICU admission, hospital mortality, and length of hospital stay. Analysis of length of stay was done using multivariable linear regression, adjusting for age, sex, and facility type, and included planned sensitivity analyses.

Results: Of the 232 cases, 77 (33.2%) were travel-related, and 155 (66.8%) were locally acquired. Most patients were male (215/232, 92.7%), with a median age of 34 years. Most of the cases were mild: 227 cases (97.8%) had mild symptoms, and there were no severe cases. No one was admitted to the ICU or died in the hospital, and all patients recovered, regardless of their travel status. Travel-related cases were a bit older (median 36 vs 33 years, p = 0.006) and more often treated in private facilities (62/77, 80.5% vs 104/155, 67.1%; p = 0.048). Travel-related cases had a longer median hospital stay (21 vs 16 days, p = 0.002). After adjusting for age, sex, and facility type, international travel was not linked to length of stay (adjusted difference −0.9 days, 95% CI −3.9 to +2.1, p = 0.55), but private facility care was linked to a longer stay (+8.5 days, p < 0.001).

Conclusions: In Jeddah city, most mpox cases were mild, with no serious outcomes or hospital mortality, regardless of travel history. Differences in hospital stay were mostly associated with the type of healthcare facility, not travel status. These findings highlight that risk assessment should focus on clinical presentation and risk factors, rather than international travel, as predictors of clinical outcomes and that ensuring proper surveillance and rapid detection is crucial.

## Introduction

Mpox (formerly known as monkeypox) is a zoonotic viral infection caused by the mpox virus. It is an orthopoxvirus related to the smallpox (variola) virus. The pathway of transmission involves transfer from animals to humans and then between humans through direct skin contact, mucosal lesions, body fluids, or contaminated materials [1]. Human cases of mpox typically show symptoms including fever, headache, myalgia, lymphadenopathy, and later vesiculo-pustular lesions that resolve within a period of two to four weeks. Severe complications include secondary bacterial infections, encephalitis, and sepsis, which can occur predominantly in immunocompromised patients [2]. Over the past decades, mpox was primarily associated with tropical rainforests of Central and West Africa, with many sporadic outbreaks and limited human-to-human transmission [3].

This pattern showed an important change in May 2022, when a multi-country outbreak of clade IIb mpox was detected across several regions designated by the World Health Organization (WHO) [2], prompting the WHO to declare a Public Health Emergency of International Concern in July 2022 [4]. A systematic review of the 2022 outbreak showed that the majority of cases were young adult males, typically characterized by mucosal and anogenital lesions. Cases were mostly associated with close sexual contact, most notably among MSM (men who have sex with men) [5]. Even though the overall mortality rate was low, a large number of patients required hospitalization for pain management or to address possible complications [2]. International travel has been identified as an important factor in the global spread of mpox outside endemic areas. Modeling studies using airline network and passenger flow data show that the likelihood of importing cases into a specific country correlates with the volume of travelers from endemic or affected areas, and that screening measures at points of entry or exit may reduce, but do not completely eliminate, the risk [6]. Robust surveillance and rapid case detection at entry points are necessary [7].

Saudi Arabia, as an important global hub for air travel and mass gatherings, particularly during the Hajj and Umrah seasons that attract millions of pilgrims and visitors from all around the world, increases the risk of imported communicable diseases, including mpox [8]. Hence, pointing out the need for early preparedness [4]. In Saudi Arabia, the first laboratory-confirmed case was reported by the Ministry of Health (MOH) on July 14, 2022. It was a case with a history of travel, suggesting international travel as the initial entry point for mpox [9]. An observational study of the first seven cases revealed all of them were adult males with recent international travel primarily to European countries and high-risk sexual exposure; all cases were mild to moderate, and all recovered without mortality [10]. On the other hand, a national study included 381 laboratory-confirmed cases between May and September 2023, primarily involving young males; the majority of cases were concentrated in Riyadh and Jeddah (~70% vs ~18%), and most cases were without international travel. Most infections were locally acquired, with very low mortality despite multiple hospital admissions [11]. Concomitantly, an outbreak investigation conducted in Jeddah identified lab-confirmed mpox cases with no documented international travel or animal exposure, supporting the hypothesis that mpox initiated local transmission within Jeddah [12].

Recent Saudi literature consists mainly of descriptive accounts and lacks rigorous comparisons of clinical outcomes by travel history [13]. To our knowledge, no published Saudi study has specifically investigated whether mpox cases with recent international travel differ from locally acquired cases in terms of clinical severity, hospitalization, or length of stay in a major gateway city such as Jeddah. Noticing these differences is important, as imported and locally acquired cases may have distinct epidemiological and clinical profiles that require customized interventions and influence decisions on risk stratification, isolation, bed capacity, and targeted health education. Accordingly, a retrospective cohort study was done to assess the impact of recent international travel on the clinical outcomes of laboratory-confirmed mpox cases in Jeddah.

## Materials and methods

Study design and setting

This is a retrospective cohort study of laboratory-confirmed mpox cases identified through the Health Electronic Surveillance Network (HESN) and hospital medical records in Jeddah, Saudi Arabia. The study was reviewed and approved by the Institutional Review Board (Approval number A02429). The study used previously collected, de-identified surveillance and hospital medical records and posed no more than minimal risk; a waiver of consent was granted.

Participants

Inclusion criteria for participants were adults (aged ≥18 years) who met the Saudi Ministry of Health definition of a confirmed mpox case (positive mpox PCR or viral isolation) [14]. The cases were reported through HESN or a hospital in Jeddah and had essential demographic, travel, and outcome data. Cases were excluded if they did not meet the confirmed-case criteria, if travel history within 21 days before symptom onset could not be determined, or if crucial clinical outcome data were missing. Duplicate records were resolved by keeping the most recent complete entry. All eligible cases during the study period were included through consecutive (census) sampling.

Exposure classification and variables

For each case, a standardized, de-identified data-extraction form was used to review the surveillance record and medical chart. The variables extracted included demographics, comorbidities, signs and symptoms at presentation, documented international travel within 21 days before symptom onset or the first positive test, reported exposures and risk behaviors, disease severity, hospitalization details, ICU admission, length of stay, and in-hospital outcome. Cases were categorized as travel-associated if documented international travel occurred within the 21-day window, or as locally acquired if no such travel occurred. The classification of cases without documented international travel as locally acquired was tested in a predefined sensitivity analysis focusing only on cases with clearly recorded travel status. The type of facility (private vs. governmental) was recorded for each case.

Statistical analysis

The distribution of continuous variables (age and length of hospital stay) was assessed using the Shapiro-Wilk test together with visual inspection of histograms and Q-Q plots. Both variables were right-skewed and departed significantly from normality (age: W = 0.968, p < 0.001; length of stay: W = 0.724, p < 0.001). Continuous variables were therefore summarized as medians and interquartile ranges (IQR) and compared with the Mann-Whitney U test; categorical variables were summarized as frequencies and percentages, n (%), and compared with Pearson's chi-square test with Yates' continuity correction, except for sex and disease severity, where Fisher's exact test was used because of small expected cell counts. Percentages for variables with incomplete documentation were calculated using the number of cases in which the variable was recorded as the denominator. Since all patients were hospitalized and had no ICU admissions, severe disease, or in-hospital mortality, the planned multivariable logistic regression analyses of adverse outcomes could not be carried out. Instead, hospital length of stay was analyzed as the primary comparable outcome using multivariable linear regression, adjusting for age, sex, and facility type; model assumptions were checked by inspection of the residual distribution and residual-versus-fitted plots. Regression coefficients are reported as adjusted mean differences in days, with 95% confidence intervals (CIs) and the corresponding t-statistics. Sensitivity analyses included (i) limiting the comparison to cases with specifically documented travel status and (ii) excluding stays longer than 45 days. All tests were two-sided, and p < 0.05 was considered statistically significant. Statistical analyses were performed using Python (version 3.12.10, Python Software Foundation, Beaverton, Oregon) with the pandas (version 2.2.3, The pandas development team, NumFOCUS, Austin, Texas) and SciPy (version 1.16.0, SciPy Community, NumFOCUS, Austin, Texas) libraries.

## Results

Study population

**Figure 1:**
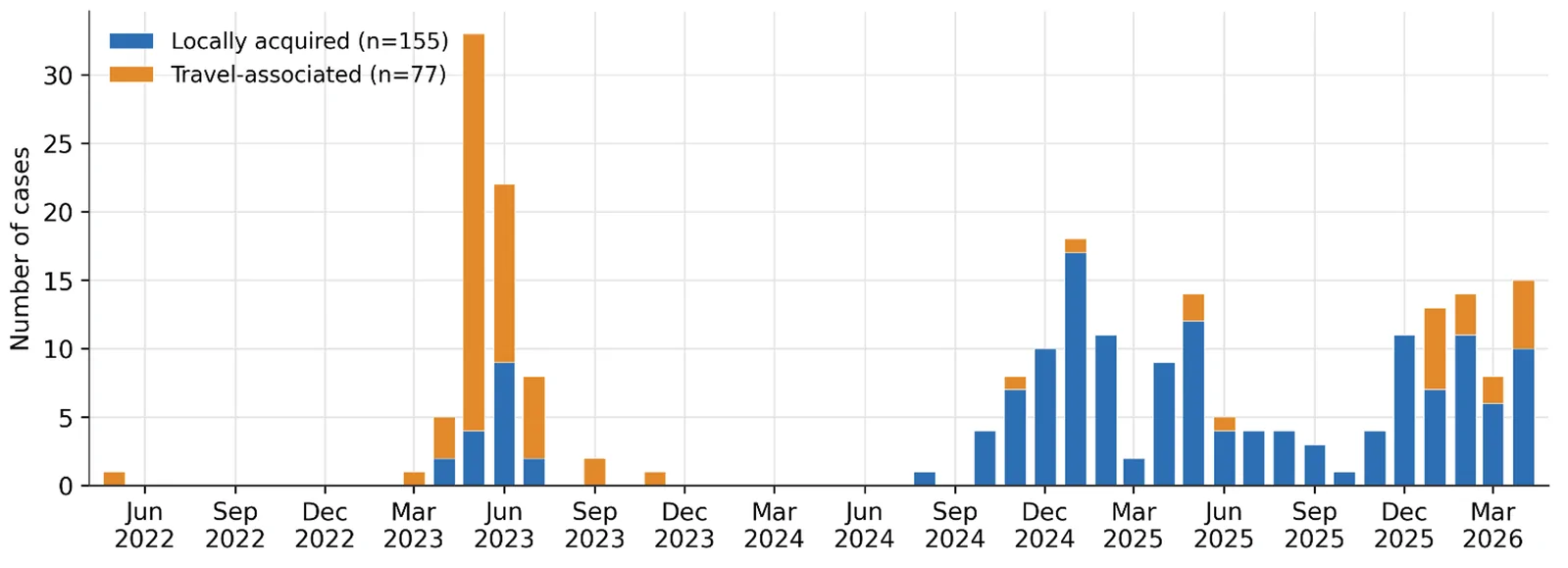
Monthly distribution of confirmed mpox cases in Jeddah by travel status (N = 232).

A total of 232 adult mpox cases confirmed by laboratory in Jeddah were analyzed. The median age was 34 years (IQR 29-41; range 18-69), with a majority being male (215/232, 92.7%). Saudi patients made up 77 cases (33.2%), while the rest were foreign residents, predominantly from Bangladesh (n = 27), Pakistan (n = 26), India (n = 19), and Yemen (n = 18). The majority of patients were reported from private hospitals (166/232, 71.6%). Travel history identified 77 cases (33.2%) as travel-related and 155 (66.8%) as locally acquired. The cases were collected between May 2022 and April 2026, with two main waves: an initial, largely travel-associated wave occurred in early 2023, followed by a larger, mostly locally acquired wave from late 2024 to 2026. (Figure 1).

Baseline and clinical characteristics

Baseline demographic, clinical, and disease-severity characteristics of the two exposure groups are summarized in Table 1. Travel-associated cases were modestly older than non-travel-associated cases (median 36 vs 33 years, p = 0.006) and were more frequently managed in private facilities (62/77, 80.5% vs 104/155, 67.1%; p = 0.048); the groups did not differ significantly by sex or Saudi nationality. Among cases with available clinical documentation, the most common presenting feature was a vesiculo-pustular rash (180/211, 85.3% overall), which was more frequently recorded among travel-associated cases than among non-travel-associated cases (75/77, 97.4% vs 105/134, 78.4%; p < 0.001). Fever (148/211, 70.1%), headache (60/209, 28.7%), myalgia (63/208, 30.3%), and lymphadenopathy (58/198, 29.3%) were similar between groups.

**Table 1:** Baseline, clinical, and outcome characteristics of confirmed mpox cases by travel status (N = 232). Data are presented as median (interquartile range [IQR]) for continuous variables and n (%) for categorical variables. Continuous variables were compared using the Mann-Whitney U test, with the U statistic reported. Categorical variables were compared using Pearson’s chi-square test with Yates’ continuity correction, with the χ² statistic and degrees of freedom reported, except for sex and disease severity, which were compared using Fisher’s exact test, for which the p value is reported. Presenting features were documented in a subset of cases; percentages were calculated using the number of cases with available data for each feature as the denominator, and the denominators are shown. All tests were two-sided, and a p value < 0.05 was considered statistically significant.

Characteristic	Overall (n = 232)	Travel-associated (n = 77)	Non-travel (n = 155)	Test statistic	p-value
Age, years, median (IQR)	34 (29–41)	36 (31–42)	33 (28–40)	U = 7281.5	0.006
Sex, n (%)				Fisher's exact	1.000
Male	215 (92.7%)	72 (93.5%)	143 (92.3%)		
Female	17 (7.3%)	5 (6.5%)	12 (7.7%)		
Saudi nationality, n (%)	77 (33.2%)	21 (27.3%)	56 (36.1%)	χ² = 1.44 (df 1)	0.230
Private facility, n (%)	166 (71.6%)	62 (80.5%)	104 (67.1%)	χ² = 3.92 (df 1)	0.048
Presenting features, n (%)*					
Vesiculo-pustular rash	180/211 (85.3%)	75/77 (97.4%)	105/134 (78.4%)	χ² = 12.67 (df 1)	<0.001
Fever	148/211 (70.1%)	55/77 (71.4%)	93/134 (69.4%)	χ² = 0.02 (df 1)	0.878
Headache	60/209 (28.7%)	28/77 (36.4%)	32/132 (24.2%)	χ² = 2.92 (df 1)	0.087
Myalgia	63/208 (30.3%)	26/76 (34.2%)	37/132 (28.0%)	χ² = 0.60 (df 1)	0.437
Lymphadenopathy	58/198 (29.3%)	21/74 (28.4%)	37/124 (29.8%)	χ² = 0.003 (df 1)	0.955
Disease severity, n (%)				Fisher's exact	1.000
Mild	227 (97.8%)	75 (97.4%)	152 (98.1%)		
Moderate	5 (2.2%)	2 (2.6%)	3 (1.9%)		
Severe	0 (0.0%)	0 (0.0%)	0 (0.0%)		
Clinical outcomes					
Hospitalised, n (%)	232 (100%)	77 (100%)	155 (100%)	—	—
ICU admission, n (%)	0 (0.0%)	0 (0.0%)	0 (0.0%)	—	—
In-hospital death, n (%)	0 (0.0%)	0 (0.0%)	0 (0.0%)	—	—
Recovered & discharged, n (%)	232 (100%)	77 (100%)	155 (100%)	—	—
Length of stay, days, median (IQR)	18 (12–21)	21 (14–21)	16 (10–20)	U = 4913.5	0.002

Disease severity and clinical outcomes

Disease was mild in most patients: 227 (97.8%) had mild illness, 5 (2.2%) had moderate illness, and none had severe illness. All 232 patients were hospitalized, and outcomes were good. No one was admitted to the ICU or died in the hospital, and everyone recovered and was discharged in both groups. Since these outcomes did not vary, the planned logistic regression analyses could not be conducted, so we focused on comparing the length of hospital stay.

Length of hospital stay

**Figure 2:**
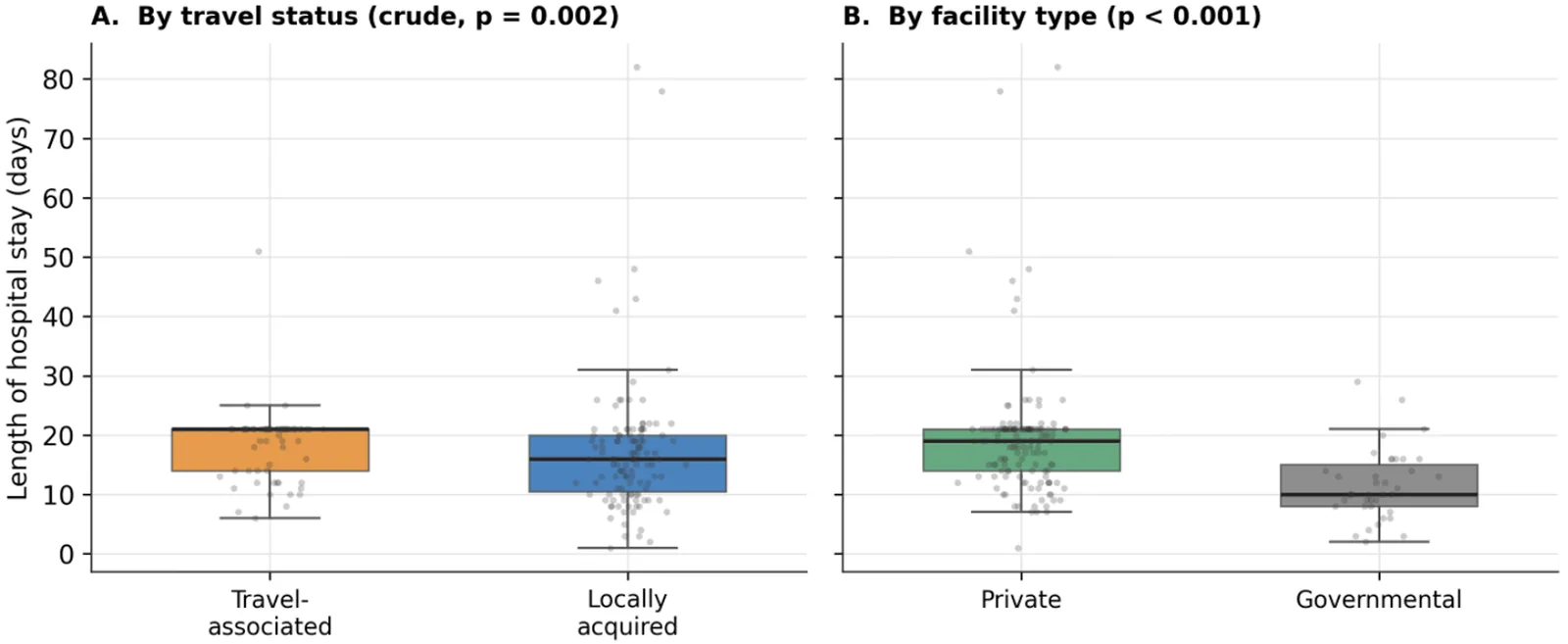
Length of hospital stay by travel status (A) and by facility type (B). Boxes show median and interquartile range; whiskers extend to 1.5×IQR; points are individual cases. The Mann-Whitney U statistics were 4913.5 for the comparison by travel status (p = 0.002) and 4543.0 for the comparison by facility type (p < 0.001).

The overall median length of stay was 18 days (IQR 12-21; available for 184 patients). In unadjusted analysis, travel-associated cases appeared to have longer stays than locally acquired cases (median 21 vs 16 days, p = 0.002; Figure 2A). However, this association was confounded by facility type: patients treated in private facilities stayed substantially longer than those in governmental facilities (median 19 vs 10 days, p < 0.001; Figure 2B), and travel-associated cases were more likely to be managed privately. After adjustment for age, sex, and facility type, international travel was not independently associated with length of stay (adjusted difference −0.92 days, 95% CI −3.94 to +2.10, p = 0.547), whereas private-facility care remained strongly associated with a longer stay (+8.45 days, 95% CI +4.93 to +11.98, p < 0.001). Regression models are presented in Table 2.

**Table 2:** Association between international travel and length of hospital stay: crude, adjusted, and sensitivity analyses. Effect estimates are reported as adjusted mean differences in days from multivariable linear regression, together with the corresponding t statistics, 95% confidence intervals, and p values, unless otherwise indicated. The crude comparison reports median length of stay and the Mann–Whitney U statistic. Analyses were performed using complete cases with available length-of-stay data (n = 184). CI, confidence interval. All tests were two-sided, and a p value < 0.05 was considered statistically significant.

Model	Effect estimate	Test statistic	95% CI	p-value
Unadjusted (crude) comparison	Median 21 vs 16 days	U = 4913.5	—	0.002
Adjusted for age + sex	+1.16 days	t = 0.75	−1.89 to +4.22	0.454
Adjusted for age + sex + facility type	−0.92 days	t = −0.60	−3.94 to +2.10	0.547
Facility type (private vs governmental)	+8.45 days	t = 4.73	+4.93 to +11.98	<0.001
Sensitivity: documented travel status only	+6.74 days	t = 5.44	+4.28 to +9.20	<0.001
Sensitivity: excluding stays >45 days (n=5)	+2.15 days	t = 2.19	+0.22 to +4.08	0.030

Sensitivity analyses

The findings remained stable throughout different alternative models. Limiting the comparison to cases with documented international travel status (77 vs 47 locally acquired) showed a longer average duration of stay among travel-associated cases (+6.74 days, 95% CI +4.28 to +9.20, p < 0.001), driven by facility type rather than travel status. The exclusion of the five prolonged stays exceeding 45 days did not alter the overall conclusion (adjusted difference of +2.15 days; 95% CI, +0.22 to +4.08, prior to facility adjustment). Throughout all analytical models, the consistent absence of severe disease manifestations, intensive care unit admissions, and mortality was observed.

## Discussion

This is the largest study from Jeddah to compare mpox outcomes by travel status. It included 232 adults with lab-confirmed mpox in Jeddah; the illness was mild in most of the cases. There were no severe cases, ICU admissions, or in-hospital mortality in either travel-related or locally acquired cases. Travel-related cases were slightly older and more often treated in private facilities, but international travel was not associated with worse outcomes. The only difference noticed, a longer hospital stay for travel-related cases, was actually due to the type of facility (governmental vs private), not travel.

Our findings match the Saudi and international experience of the 2022 clade IIb outbreak. The initial cases in Saudi Arabia were travel-related, mostly affected adult men, often involved high-risk exposure, and were consistently mild with full recoveries [10]. A later national cohort of 381 cases also showed a young, predominantly male population with low mortality despite repeated hospitalizations [11], and an outbreak investigation in Jeddah found locally acquired cases with no international travel history or animal contact, indicating community transmission within the city [12].

The high proportion of male patients (215/232, 92.7%) and the median age of 34 years in our cohort are consistent with these data and the global epidemiology of the 2022 outbreak [4]. Our data add to this evidence by demonstrating that, once cases reach care in Jeddah, clinical outcomes are similar for imported and locally acquired infections. The two-wave pattern (Figure 1), i.e., an initial travel-related wave followed by a larger, locally transmitted wave, is consistent with a shift from importation to sustained community transmission.

The main finding of our study is about both methods and clinical care. The length of hospital stay was the only outcome that varied, and it was initially longer in travel-related cases. However, this difference went away after we adjusted for facility type. Patients in private care stayed 8.5 days longer. This means that hospital stay is affected more by the health system and administrative factors than by how severe the disease is or how it was detected. So, the length of stay should be used carefully as an outcome in mpox studies, since not adjusting for health system factors can give the wrong impression about patient exposures. From a public-health perspective, these outcomes have two main implications. First, the mild disease and lack of severe results, regardless of travel history, suggest that using travel history alone for triage is inadequate; risk should be based on host clinical presentation and risk factors, such as immunosuppression, which drive severe mpox [1,2,5]. Second, with many cases having no documented international travel and local transmission confirmed in Jeddah [12], preparedness must go beyond border measures [15]. While surveillance and rapid detection at entry points are vital, along with community surveillance, contact tracing, and targeted health education for high-risk groups are similarly essential.

Strengths and limitations

The main strengths of this study are its size: it is the largest mpox group reported from Jeddah to date, and its use of routine surveillance and hospital data from multiple facilities, providing a real-world view of mpox care in a major city. The analysis plan was set in advance, and several sensitivity checks supported the main results.

There are several limitations to note. This was a retrospective study based on hospital records and data from the health electronic surveillance network, and the quality of documentation varied. These records sometimes did not consistently record key details like immune status, HIV status, exposures, or risk behaviors, so the true rates of symptoms and signs may be underestimated, and we could not fully adjust for these factors. Most missing data were in locally acquired cases, but a sensitivity analysis using only cases with documented travel confirmed our main findings. Also, in some hospitals' records, clinicians may miss the 21-day frame or mix up international vs domestic travel. Finally, since all patients were hospitalized and none had severe illness or in-hospital mortality, we could not do the planned logistic regression analyses, and our results may not represent mild outpatient cases or apply to other parts of Saudi Arabia. But we highlight that risk assessment should be more focused on clinical presentation and risk factors rather than travel status as predictors for clinical outcomes, while ensuring proper surveillance and early, rapid detection is crucial.

## Conclusions

In 232 adults with confirmed mpox in Jeddah, the disease was mostly mild, with no serious outcomes or deaths, and international travel was not linked to worse outcomes. Differences in hospital stay were due to facility type, not travel. These findings support using patient and clinical factors, not just travel history, for risk assessment, along with both entry-point and community surveillance, since mpox is now spreading locally in Jeddah. For better future surveillance and research, clinicians should carefully document all parts of the clinical case definition, including a clear travel history with exact dates and destinations, and note whether travel was international or domestic within the 21-day window. Entering this information accurately into the HESN system will improve case classification and allow for better studies of mpox outcomes in the future.
